# Aortic valve calcium score in hypercholesterolemic patients with and without low-density lipoprotein receptor gene mutation

**DOI:** 10.1371/journal.pone.0209229

**Published:** 2018-12-28

**Authors:** Rafal Gałąska, Dorota Kulawiak-Gałąska, Magdalena Chmara, Krzysztof Chlebus, Michał Studniarek, Marcin Fijałkowski, Bartosz Wasąg, Andrzej Rynkiewicz, Marcin Gruchała

**Affiliations:** 1 1st Department of Cardiology, Medical University of Gdansk, Gdansk, Poland; 2 Department of Radiology, Medical University of Gdansk, Gdansk, Poland; 3 Department of Biology and Genetics, Medical University of Gdansk, Gdansk, Poland; 4 Department of Cardiology and Cardiosurgery, University of Warmia and Mazury, Olsztyn, Poland; Beijing Key Laboratory of Diabetes Prevention and Research, CHINA

## Abstract

The aim of this study was a comparison of aortic valve calcium score (AVCS) between patients with hypercholesterolemia and genetic diagnosis of familial hypercholesterolemia with low-density lipoprotein receptor gene mutation (LDLR-M group), versus patients with hypercholesterolemia without LDLR gene mutation (LDLR-WT group). A total of 72 LDLR-M patients and 50 LDLR-WT patients were enrolled in the study and underwent CT as a part of an assessment of coronary calcium scoring. AVCS was determined and compared between the two patient groups. AVCS was significantly higher in the LDLR-M group in comparison to the LDLR-WT group (13.8 ± 37.9 vs. 0.94 ± 3.1, p = 0.03). The Yates' chi-squared test for independence revealed that *LDLR* mutation and AVCS were significantly dependable (Chi^2 = 6.106, p = 0.013). The *LDLR* mutation was a strong predictor of a high AVCS (OR 7.83, 95% CI 2.08–29.50, p = 0.002) on multivariate regression analysis. Among the traditional risk factors, age (odds ratio 1.12, 95% CI 1.05–1.18, p<0.001) and SBP (OR 1.04, 95% CI 1.00–1.07, p = 0.045) were also significant for high result of AVCS. An assessment of computed tomography calcium scores showed that LDLR-M patients have increased AVCS in comparison to those with LDLR-WT. In addition, *LDLR* mutation can be considered as an independent risk factor of having high AVSC even after adjustment for risk factors including cholesterol levels. This may result from the associated process connected with the regulatory role of LDLR in evolution of aortic valve calcifications.

## Introduction

Familial hypercholesterolemia (FH) is an autosomal dominant disorder, characterized by high cholesterol levels, which can be caused by a mutation in the low-density lipoprotein receptor (LDLR) gene. Early presence of coronary artery disease (CAD), as well as calcific aortic stenosis (CAS) is the major complication of untreated FH. CAS is the third-leading cause of cardiovascular disease [1]. As previously reported, it affects approximately 3% of adults over 75 years [2]. CAS and CAD may result from different pathogeneses and molecular mechanisms.

CAS has been considered to be a 'degenerative' disease and part of the aging process, however, some evidence suggests that CAS is a result of an active pathological process with some similarities to atherosclerosis. Risk factors such as age, male gender, hypertension, hypercholesterolemia, diabetes, and smoking are common to both processes [3] but have been found to be relatively poor predictors of CAS. Furthermore, the exact role of hypercholesterolemia in the pathogenesis of aortic stenosis remains unclear.

Recent data suggest that additional factors such as osteogenic processes may contribute to the onset and progression of CAS [4]. It has been suggested that Wnt signaling pathways, which play a fundamental role in the differentiation, proliferation, and death of many cells can be responsible for osteoblast differentiation in the aortic valve [5], thus promoting calcification. Control of Wnt pathways is modulated by a number of proteins including LDLR-related protein (LRP) [5,6]. LRP5 is an LDLR coreceptor involved in activation of skeletal bone formation and also implicated in cholesterol metabolism. It is expressed in low concentration in many tissues including the aortic valve [6]. There is growing experimental evidence that at least in part, the evolution of valvular calcifications appears to be independent of LDL-C levels and physiological signaling pathways responsible for this process can be dysregulated in LDLR mutation patients. Accurate quantification of aortic valve calcifications is possible by aortic calcium score assessment using cardiac computed tomography.

The aim of this study was to compare aortic valve calcium score (AVCS) between patients with severe hypercholesterolemia and DNA-based diagnosis of familial hypercholesterolemia with confirmed *LDLR* mutation (LDLR-M group) versus patients with non-familial severe hypercholesterolemia with no LDLR alteration (LDLR-WT group). Furthermore, we aimed to assess risk factors, which correlated with a high result of AVCS in both groups.

## Methods

### General information

The study group was selected from patients who had clinical suspicion of FH and who were admitted to our outpatient preventive clinic between 2010 and 2013. For all FH patients having at least 3 points according to the Dutch Lipid Clinic Network, genomic DNA was isolated from their whole blood using standard methods and mutational analysis of LDLR and APOB was performed as previously described [7,8]_,_ We evaluated a total of 156 adult subjects with a DNA-based diagnosis of FH. Only those with confirmed *LDLR* mutation, with age 30 years or more and with no previous history of clinically apparent cardiac disease were included in the analysis. Other exclusion criteria were: secondary hypercholesterolemia due to thyroid or liver disease, renal insufficiency (estimated creatinine clearance <50 ml/min), TG level 4,5 mmol/l (>400 mg/dl), and pregnancy. Finally, 72 patients (32 men and 40 women) were included, with a mean age of 49.6 ± 12 years. AVCS assessment was also performed on a group of 50 randomly selected individuals who were diagnosed with severe hypercholesterolemia during the same period with nonconfirmed FH causing mutation (LDLR-WT group) and with an LDL-C level above 4,9 mmol/L (190 mg%). The remaining inclusion and exclusion criteria were the same as for the study group. Both groups did not differ significantly in terms of mean age and sex ratio.

### Aortic valve calcium score determination

**Fig 1:**
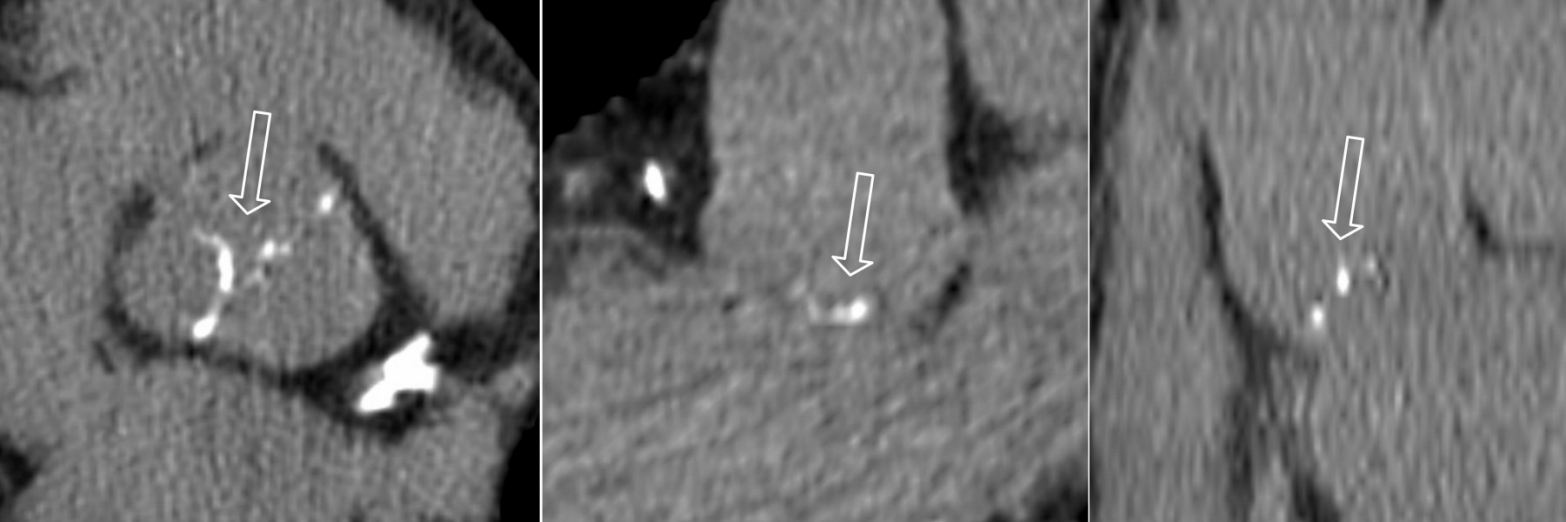
Computed tomography multiplanar refortmats in 3 orthogonal planes confirming the location of the aortic valve calcifications (arrows).

**Fig 2:**
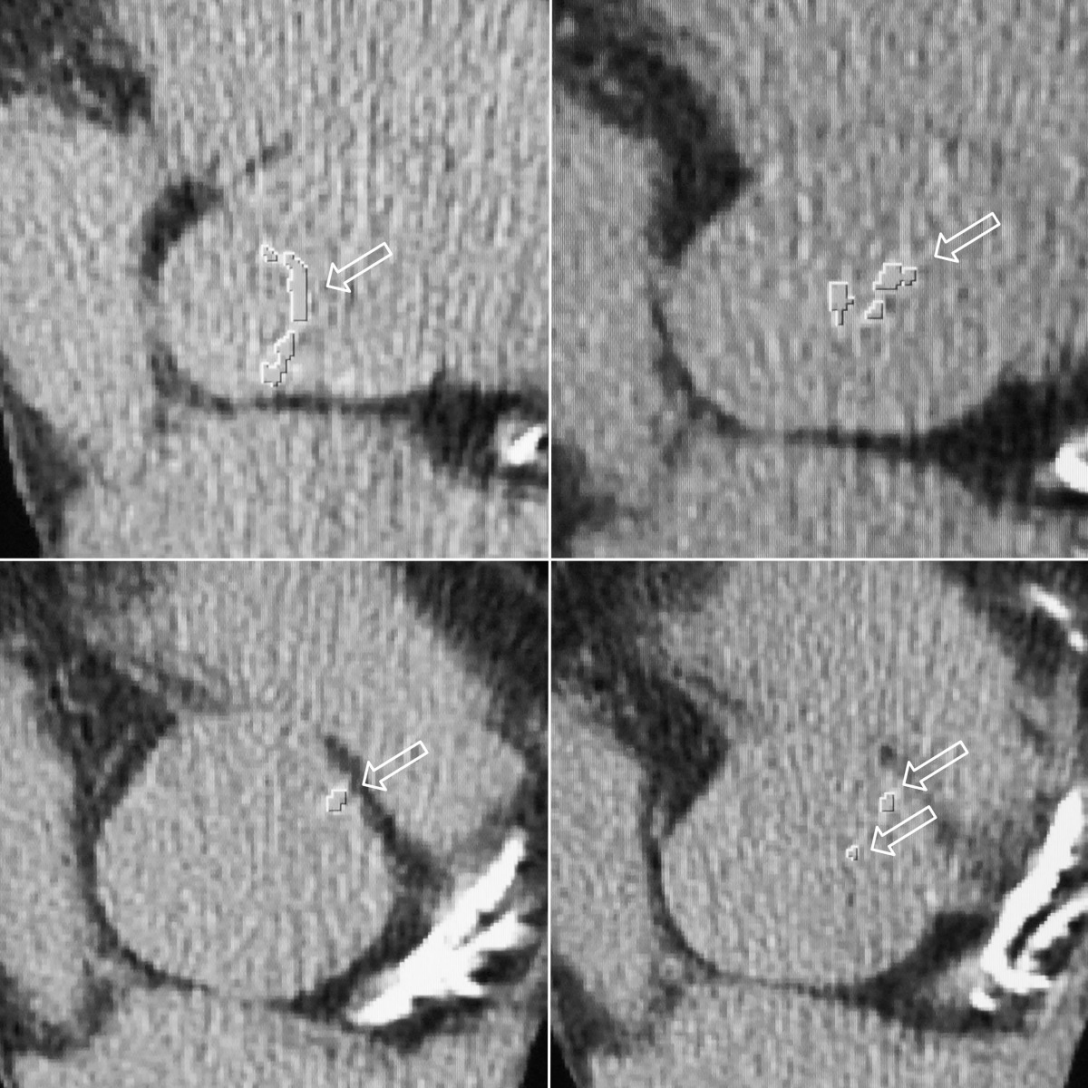
Computed tomography axial scans illustrating aortic valve calcifications indentified by means of SmartScore software (arrows).

After signing a written informed consent, cardiac ECG-gated computed tomography (CT) was performed on all included patients. The local ethics committee approved the study protocol. Computed tomography was performed during inspiration, scanning from the aortic arch to the diaphragm using a 64 row CT scanner (GE) with ECG gating. A prospective electrocardiogram-triggered scan protocol at 70% of the RR-interval was used. Images were reconstructed with a slice thickness of 1.25 mm. Only calcifications located within aortic valve leaflets with attenuation value >130 HU were used to obtain AVCS (S1 Fig). The location of the aortic valve calcifications was verified using multiplanar refortmats in the three orthogonal planes (Fig 1). A radiologist with five years’ experience in calcium scoring, who was blinded to all clinical data calculated AVCS for each patient using volumetric methods on the axial CT scans by means of SmartScore 4.0 software (Fig 2). AVCS was defined as sum of all partial values of aortic valve calcifications in a patient and expressed in mm^3^.

### Clinical measurements

Current lipids levels were obtained from each patient by standard methods in a central clinic laboratory. Patients fasted for at least 12 hours prior to blood sampling. Thus, for patients without previous lipid lowering treatment, maximum and current cholesterol values were the same. If patients were on lipid lowering treatment their highest cholesterol values before treatment were taken into account. Since discontinuation of lipid lowering drugs in patients with severe hypercholesterolemia was not an option we analyzed previous lipid results from various laboratories including central laboratory. Diabetes was defined as a fasting blood glucose level greater than 7 mmol/L or use of hypoglycemic medications. Smoking history was defined as positive for former or present smokers who had smoked more than 1 pack-year.

### Statistical analysis

Continuous variables are shown as means and standard deviation except for calcium score results, which are also shown as median and quartile values. Categorical variables are expressed by number and percentages. Data normality was tested using the Kolmogorov–Smirnov test. The data sets were compared using the two-sided t-test or Mann-Whitney U-test for non-parametric variables depending on the variables distribution. Categorical variables were compared with Fisher’s exact test. The correlations between those variables was tested by Pearson’s correlation coefficients or chi^2 independence test. In order to determine the best set of variables explaining dichotomous variable AVCS we decided to use multivariate regression analysis (see results section for details). The analyses were performed with Statistica 10 software. A p value < 0.05 was considered statistically significant.

## Results

**Fig 3:**
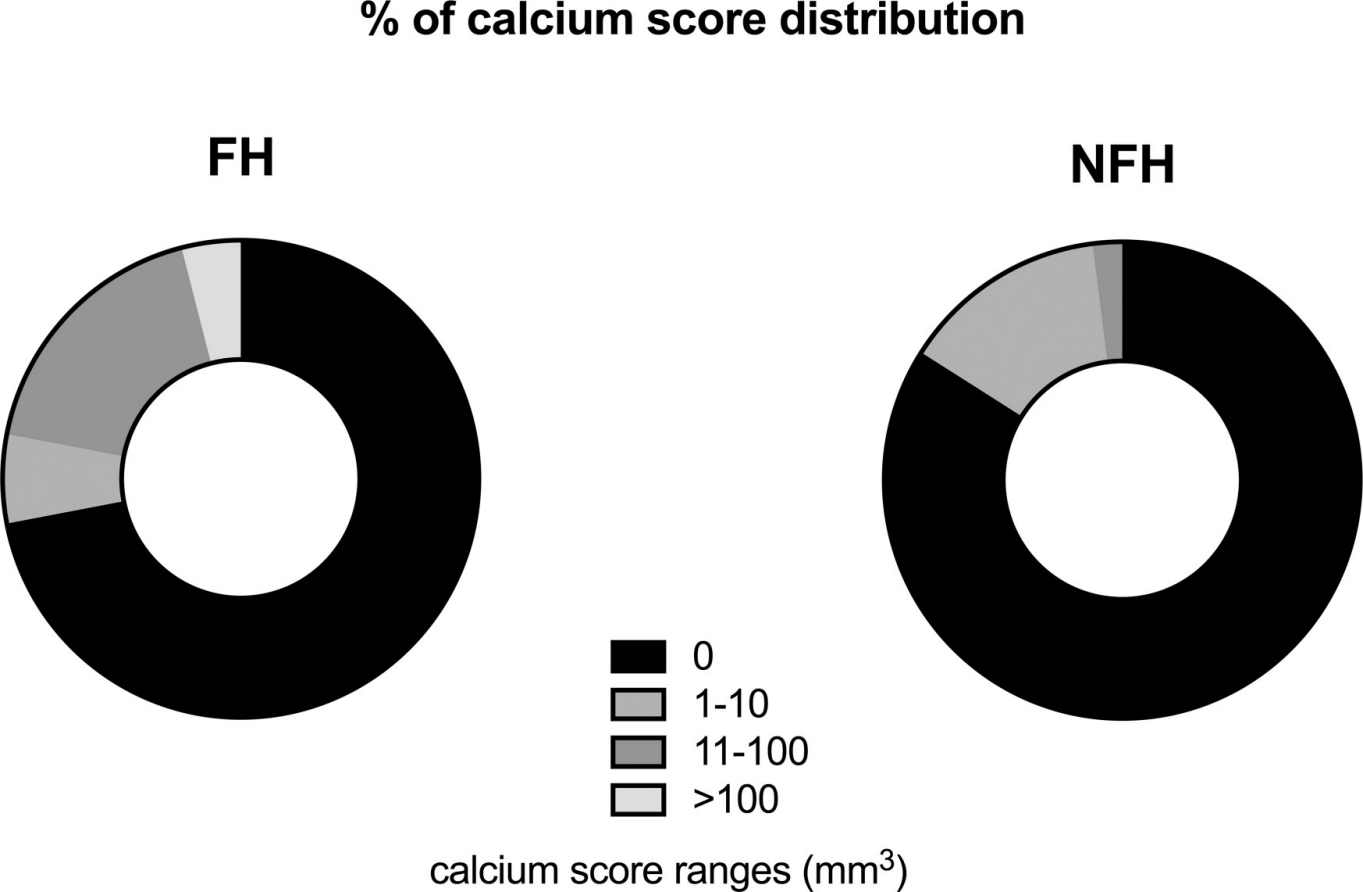
Calcium score distribution across AVCS in LDLR-M and LDLR-WT groups. Data in particular ranges are expressed as % of each calcium score subgroup. **Abbreviations:** LDLR-M–patients with familial hypercholesterolemia and LDLR mutation, LDLR-WT–nonfamilial hypercholesterolemia, AVCS—aortic valve calcium score.

Clinical characteristics of the 72 LDLR-M and 50 LDLR-WT patients are presented in Table 1. We found no statistical difference in body mass index (BMI), history of diabetes and hypertension, or triglycerides (TG) levels between the LDLR-M and LDLR-WT groups. However, the LDLR-M group had higher maximum total cholesterol (TCmax) and LDL-C max levels and lower on-treatment high-density lipoprotein cholesterol (HDL-C) levels. The most frequent *LDLR* alteration in LDLR-M group was the c.662A>G point mutation resulting in p.D221G substitution, which was detected in 14 of the patients. In 8 of the patients, the p.G592E mutation was observed, whereas major reararrangements and frameshift or nonsense mutations were found in 8 of the individuals. In 23 of the patients, other missense mutations (p.G20R, p.C34G, p.C89R, p.D168G, p.S177L, p.F282L, p.G373C, p.N564S, and p.P608T) were found. Furthermore, 16 intronic variants were detected, some of which might be of substantial importance for LDL receptor activity. We found also the coexistence of *LDLR* and *APOB* alterations in three individuals, which was a heterozygous substitution at codon 3527 of *APOB*. The results of AVCS calculated using volumetric methods including AVCS distribution across calcium score and lipid levels are presented in Table 2, Fig 3 and S2 Fig.

**Table 1:** Clinical characteristics of LDLR-M and LDLR-WT groups.

	**LDLR-M (N = 72)**	**LDLR-WT (N = 50)**	**p**
Age (years)	49.1 ± 11.9	51.5 ± 9.9	ns
Gender	32 M, 40 F	24 M, 26 F	ns
BMI (kg/m^2^)	26.5 ± 4.2	26.9 ± 4.1	ns
TCmax (mmol/L)	9.6± 2	8.2 ± 1.0	<0.001
LDLmax (mmol/L)	7.3±1.7	5.9 ± 1.0	<0.001
HDLmax (mmol/L)	1.5±0.4	1.6 ± 0.3	ns
TGmax (mmol/L)	1.6±0.9	1.6 ± 0.9	ns
TCYscore (mmol-year/L)	450.1 ± 143.8	423.1 ± 114.3	ns
TC (mmol/L)	7.4±2.3	7.4 ± 1.5	ns
LDL (mmol/L)	5.4±2.1	5.1 ± 1.3	ns
HDL (mmol/L)	1.4±0.3	1.5 ± 0.3	= 0.04
TG (mmol/L)	1.4±0.7	1.5 ± 0.7	ns
SBP (mmHg)	131.9 ± 15.5	133.4 ± 14.5	ns
DBP (mmHg)	83 ± 11.1	82.7 ± 8.7	ns
Diabetes^a^ (n)	1 (1.4%)	5 (10%)	ns
Smoking^b^ (n)	24 (33.3%)	21 (42%)	ns
Statin treatment on 1^st^ visit (n)	38 (52.8)	20 (40%)	ns

**Abbreviations:** LDLR-M–patients with hypercholesterolemia and confirmed LDLR mutation, LDLR-WT (LDLR-wild type)–patients with hypercholesterolemia and nonconfirmed LDLR-mutation, NS–not significant, BMI—body mass index, TC and TCmax–total cholesterol level during inclusion to the study and maximum level without pharmacotherapy (same values for patients without prior lipid lowering treatment), LDL and LDLmax—low‐density lipoprotein, HDL and HDLmax—high‐density lipoprotein cholesterol, TG and TGmax–triglycerides, TYCscore–total cholesterol year score, SBP–systolic blood pressure. DBP–diastolic blood pressure

^a^treatment with insulin or oral anti-diabetic medicine^,^

^**b**^smoking—ever, > 1 pack-year.

**Table 2:** Volumetric calcium score of aortic valve in LDLR-M and LDLR-WT groups.

		LDLR-M	LDLR-WT	P
**AVCS (mm**^**3**^**)**	Median	0	0	P = 0.03
	mean ± SD	13.8 ± 37.9	1.06 ± 3.2	
	Q1	0	0	
	Q3	9.5	0	
	Max	196	19	
	Min	0	0	

**Abbreviations:** LDLR-M–patients with familial hypercholesterolemia and LDLR mutation, LDLR-WT–nonfamilial hypercholesterolemia, AVCS—aortic valve calcium score, NS–not significant, SD–standard deviation, Q1 –first quartile, Q3 –third quartile

**Fig 4:**
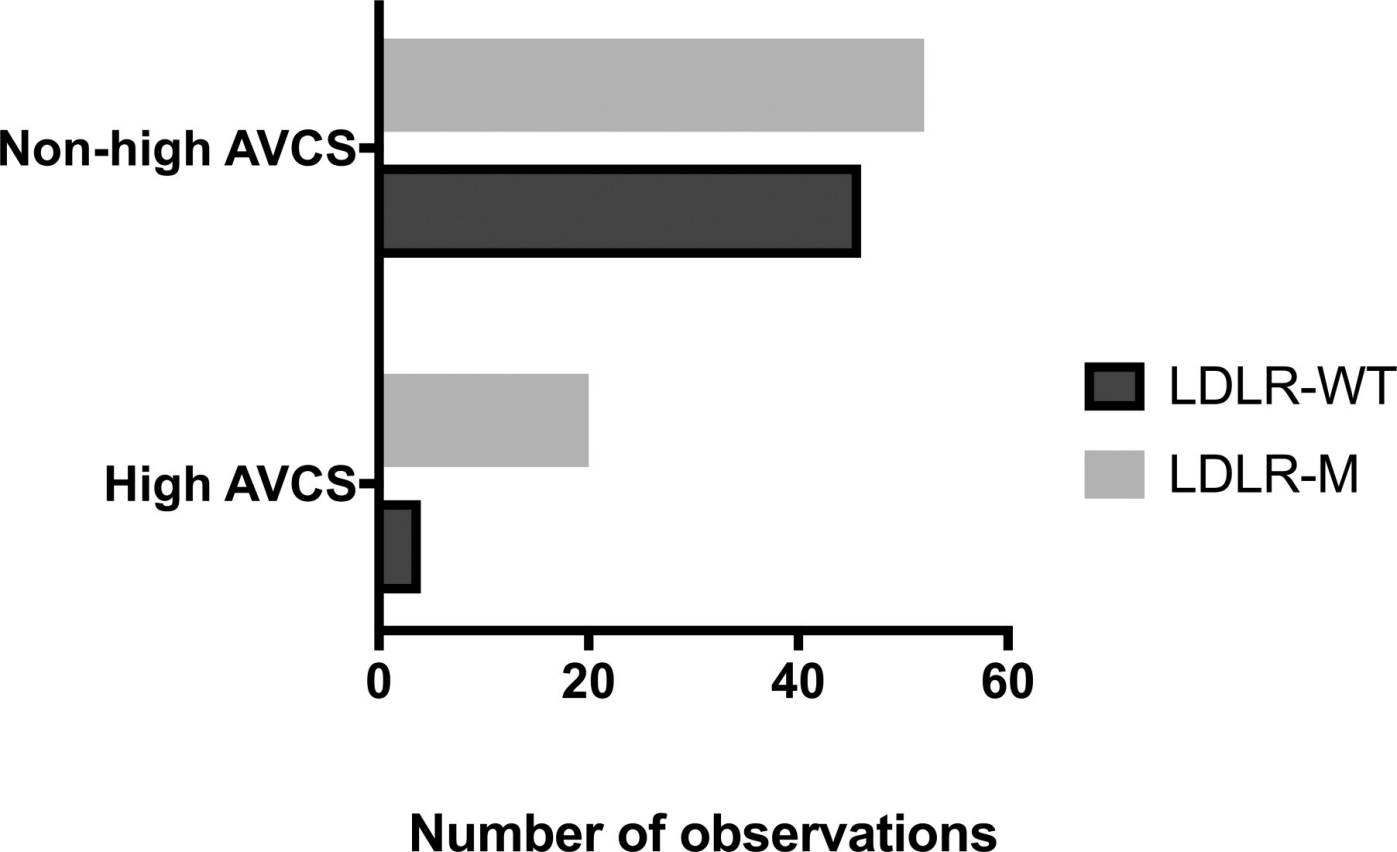
Histogram of the contingency table of patients with high and non-high AVCS. **Abbreviations:** LDLR-M–patients with familial hypercholesterolemia and LDLR mutation, LDLR-WT–nonfamilial hypercholesterolemia, high AVCS–patients with high aortic valve calcium score, non-high AVCS–remaining patients.

We found AVCS to be significantly higher in the LDLR-M group in comparison with the LDLR-WT group. We sought to determine whether the FH mutation was connected with higher results of calcium scores. For this analysis, we selected patients with high AVCS results if the value was in the top 20% of the calcium score distribution. The Yates' chi-squared test for independence revealed that *LDLR* mutation and AVCS were significantly dependable (Chi^2 = 6.106, p = 0.013, Fig 4). To determine the best set of variables that explains dichotomous variable AVCS multivariate regression, analysis was applied with backward stepwise selection. Among all the considered variables (S1 Table) only age, TCmax, LDLmax, TGmax, TCYscore, *LDLR* mutation prevalence, SBP and DBP differed significantly in the high and non-high AVCS subgroups and were used in further analysis. The correlations between variables were tested by Pearson’s correlation coefficients or chi^2 independence test (results not presented). Finally, the presented model took into account those independent variables that were strongly correlated with AVCS but not with each other (Table 3). The chosen estimation method for parameters used in the model was Quasi-Newton’s algorithm. As a result of multivariate logistic regression analysis, a positive value of LDLR mutation was a strong predictor of the high AVCS (OR 7.83, 95% CI 2.08–29.50, p = 0.002). Among the traditional risk factors, age (odds ratio 1.12, 95% CI 1.05–1.18, p<0.001) and SBP (OR 1.04, 95% CI 1.00–1.07, p = 0.045) were significant for high AVCS.

**Table 3:** Multivariate logistic regression analysis for identification of patients with high AVCS (N = 122, high AVCS N = 24).

N = 122	Chi2 = 35.63, p < 0.001			
	Constant	Age	LDLR-M	SBP
**Wald**	19.78	13.98	9.45	4.01
**Odds ratio**		1.12	7.83	1.04
**-95%CI**		1.05	2.08	1.00
**+95%CI**		1.18	29.50	1.07
**p**	<0.001	<0.001	0.002	0.045

**Abbreviations:** AVCS–aortic valve calcium score, SBP–systolic blood pressure, LDLR-M—LDL receptor mutation

## Discussion

We found that the presence of LDLR mutation was connected with higher AVCS in the group of LDLR-M patients in comparison with the group of LDLR-WT patients. The association of CAS with hyperlipidemia is well documented, however previous reports focused on the comparison of AVCS between FH patients and control subjects with normal cholesterol levels [9, 10, 11]. In our study, both groups had severe hypercholesterolemia. A variety of transcription factors, inflammatory stimuli and metabolites are responsible for the development of aortic stenosis [12,13]. According to the Cardiovascular Health Study, independent clinical factors associated with degenerative aortic valve disease include age, with a twofold increased risk for each 10-year increase in age, male gender with a twofold increase in risk, continued smoking with a 35% increase in risk, and a history of hypertension with a 20% increase in risk. LDL-C was also an independent factor in aortic stenosis but with a relatively small odds ratio of 1.1 associated with increases in LDL-C from the 25^th^ to 75^th^ percentile [3]. Severe aorta and aortic valve calcifications were reported in patients with both homozygous and heterozygous FH.

Rallidis et al. found a significant positive correlation between aortic valve root score based on mobility and thickness of aortic valve leaflets in echocardiography and total cholesterol-year score (CYS) in the FH heterozygotes aged < 70 years. On multivariate analysis, TC and LDL-C levels at diagnosis and CYS were the main predictors of aortic valve thickening. They also observed that homozygote patients with FH get severe aortic root and aortic valve calcifications at lower CYS than heterozygotes. In their opinion, it is possible that maximum cholesterol level rather than total cholesterol burden plays an important role in development of atherosclerotic changes in the aortic root [14]. Based on the association of CAS with hyperlipidemia and atherosclerosis, clinical trials of statins were undertaken [15, 16, 17, 18, 19]_._ The results of meta-analysis showed that statin therapy did not reduce aortic stenosis progression despite significant reduction in LDL-C levels [20].

In our study, age, TC, LDL-C, TCYscore, SBP and DBP were significantly higher in the subgroup with high AVCS in comparison to the remaining patients. Using multivariate regression analysis, only *LDLR* mutation, age and SBP remained independent risk factors of having high AVCS. In recent years, the exact role of hypercholesterolemia in formation of aortic valve calcifications has become a matter of debate. Using an experimental model of hypercholesterolemia, Awan et al. did not find any correlation between aortic valve calcification severity and cholesterol levels. They reported that the degree of aortic calcification in computed microtomography was over 70 times more in chow fed C57Bl/6 Ldlr-/- mice than in WT mice fed a high-cholesterol diet, even though the latter exhibited higher total cholesterol levels (440±151mg/dL versus 316±65mg/dL) [21]. In another study, Awan et al. noticed a strong correlation between aortic calcification score and age (r = 0.73, p = 0.0001), but not with total cholesterol (r = -0.36, p = 0.07) in patients with homozygous mutation in whom aortic calcification was determined by computed tomographic (CT) scan [22]. Alrasadi et al. reported in patients with heterozygote mutation that pretreatment TC had little correlation with the aortic calcium score (r = 0.51, P = 0.0435) [23].

All these data suggest that the role of LDLR in formation of vascular calcification can be connected with other mechanisms, not only those related to lipid levels [2]. LDLR may play an important role in regulation of the osteogenic signaling pathway process due to its similarity to LRP1, LRP5 and LRP6 proteins (low-density lipoprotein receptor-related proteins) which belong to the LDLR superfamily of proteins [2]. The possible cross-talk between LDR and LRP may promote osteoblast differentiation and bone formation in case of complete or partial absence of LDLR. Avan et al, observed an increased expression of LRP5 in the aorta’s subintimal layer, as well as within the tunica media in Ldlr−/− mice compared with age-matched controls on the same diet [21].

According to Fantus et al, the calcification process may proceed independently of cholesterol levels once subendothelial damage has occurred [2]. Statins may slow down or even regress the process of atherosclerosis, but they do not stop the calcification process after osteoblast-like cells differentiation. This hypothesis is supported by the findings of Messika-Zeitoun et al. who found that progression of calcification in patients with established calcific aortic valve disease is fastest in patients with highest baseline calcification score. That progress seems to be unrelated to cardiovascular risk factors based on results of calcium score after a follow-up period [24]. Our results confirm previous findings, that calcific-aortic valve disease cannot be fully explained based solely on cholesterol levels and non-lipid mechanisms can be involved in progression of CAS.

In our analysis, *LDLR* mutation was a strong predictor of high AVCS, but not cholesterol level. Of course, the presence of LDLR mutation may be a reflection of higher cumulative exposure burden of cholesterol in LDLR-M patients when compared with LDLR-WT patients. Nevertheless, based on our results, it is superior in predicting the existence of severe aortic valve calcifications in comparison with pretreatment and on-treatment measurements of cholesterol levels.

### Limitations of the study

The main limitation of this single-center study is the relatively small number of patients. Our model met all necessary requirements of multivariate logistic regression including random sample selection, number of observations, lack of collinearity of independent variables and normality of residuals, however, the small numbers might have overfitted the multivariable analysis. Moreover, many of the patients with severe hypercholesterolemia enrolled in the study had been treated for a long period with statins. This could have affected the natural history of formation of the calcifications. The influence of total cholesterol burden on aortic valve calcifications is evaluated based on two measurements, those taken without treatment and those with treatment. This may not accurately reflect the changes in cholesterol levels through life, especially in childhood and adolescence. Therefore, the predictive value of cholesterol levels both before and during treatment, as well as total cholesterol year score of AVCS may be of limited value. Further research is needed to confirm our preliminary results.

## Conclusions

FH patients with LDLR mutation have an increased AVCS, as assessed by computed tomography calcium scores, in comparison to patients with non-familial severe hypercholesterolemia. LDLR mutation is an independent risk factor of having high AVCS, even after adjustment for risk factors including cholesterol levels. These findings confirm that at least in part, the evolution of aortic valve calcifications in patients with familial hypercholesterolemia appears to be independent of cholesterol levels and may result from associated processes connected with the regulatory role of LDLR in osteogenic signaling pathway.

## Supporting information

S1 Fig Schematic representation of the aortic valve in two orthogonal planes. Only calcifications located within aortic valve leaflets were used to obtain AVCS (dashed line, grey area).
Abbreviations: AVCS—Aortic valve calcium score.
(TIFF)

S2 Fig AVCS, cholesterol and triglycerides levels scatter plots. Abbreviations: AVCS—Aortic valve calcium score, LDLR-M–patients with hypercholesterolemia and confirmed LDLR mutation, LDLR-WT (LDLR-wild type)–patients with hypercholesterolemia and nonconfirmed LDLR-mutation, TCmax–total maximum cholesterol level without pharmacotherapy, LDLmax—low‐density lipoprotein, HDLmax—high‐density lipoprotein cholesterol, TGmax–triglycerides.
(TIFF)

S1 Table Clinical characteristics of high and non-high AVSCS subgroups. **Abbreviations**: LDLR-M–patients with hypercholesterolemia and confirmed LDLR mutation, NS–not significant, BMI—body mass index, TC and TCmax–total cholesterol level during inclusion to the study and maximum level without pharmacotherapy (same values for patients without prior lipid lowering treatment), LDL and LDLmax—low‐density lipoprotein, HDL and HDLmax—high‐density lipoprotein cholesterol, TG and TGmax–triglycerides, TYCscore–total cholesterol year score, SBP–systolic blood pressure. DBP–diastolic blood pressure. ^a^treatment with insulin or oral anti-diabetic medicine. ^**b**^smoking—ever, > 1 pack-year, AVCS—Aortic valve calcium score.
(DOCX)
