# Supplementary figures and images for: Aortic valve calcium score in hypercholesterolemic patients with and without low-density lipoprotein receptor gene mutation
Source: PLoS One. 2018 Dec 28;13(12):e0209229. doi: 10.1371/journal.pone.0209229 (PMC6310281; doi:10.1371/journal.pone.0209229)

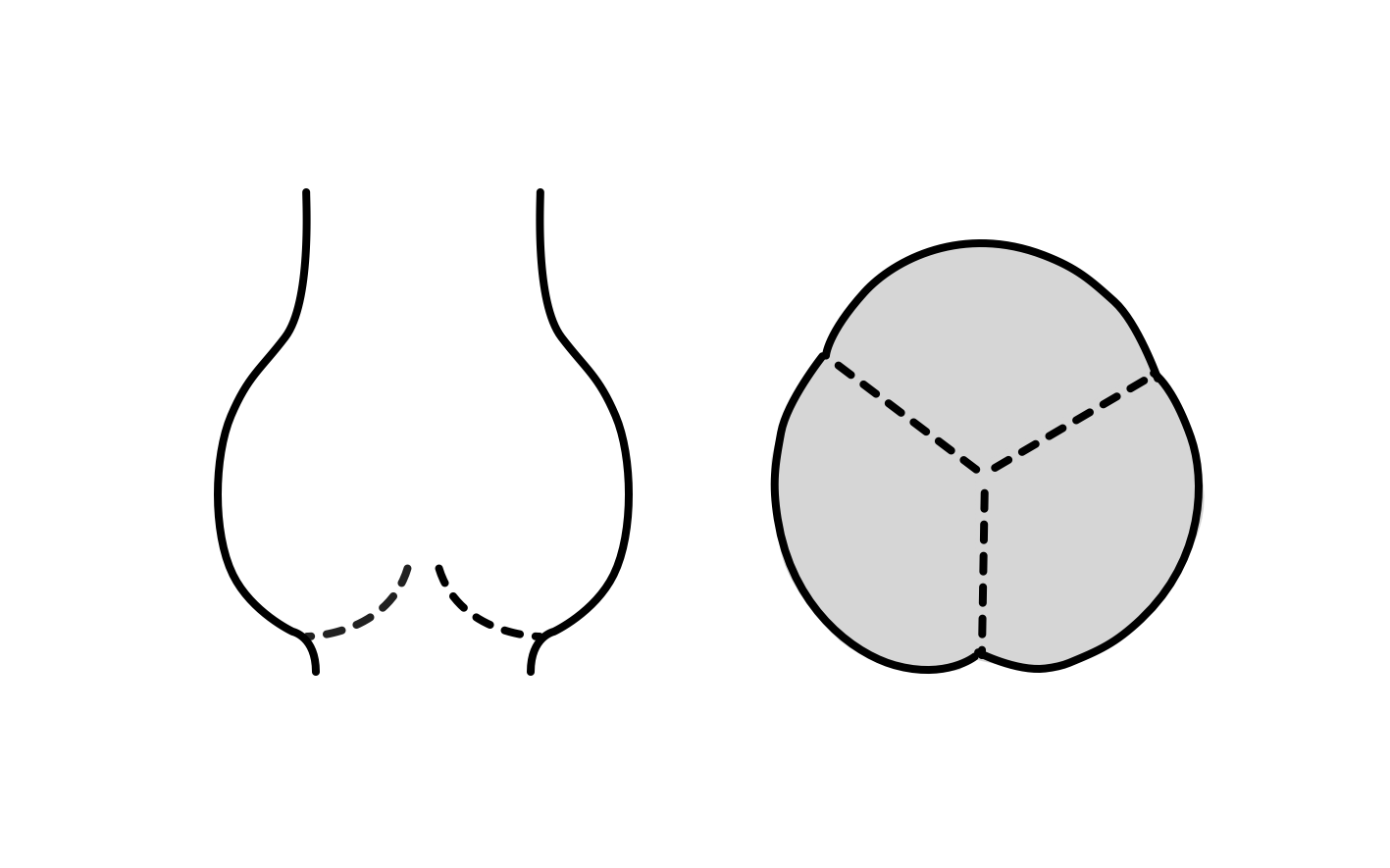

Supplement: S1 Fig — Only calcifications located within aortic valve leaflets were used to obtain AVCS (dashed line, grey area). Abbreviations: AVCS—Aortic valve calcium score. (TIFF) [file pone.0209229.s001.tiff]

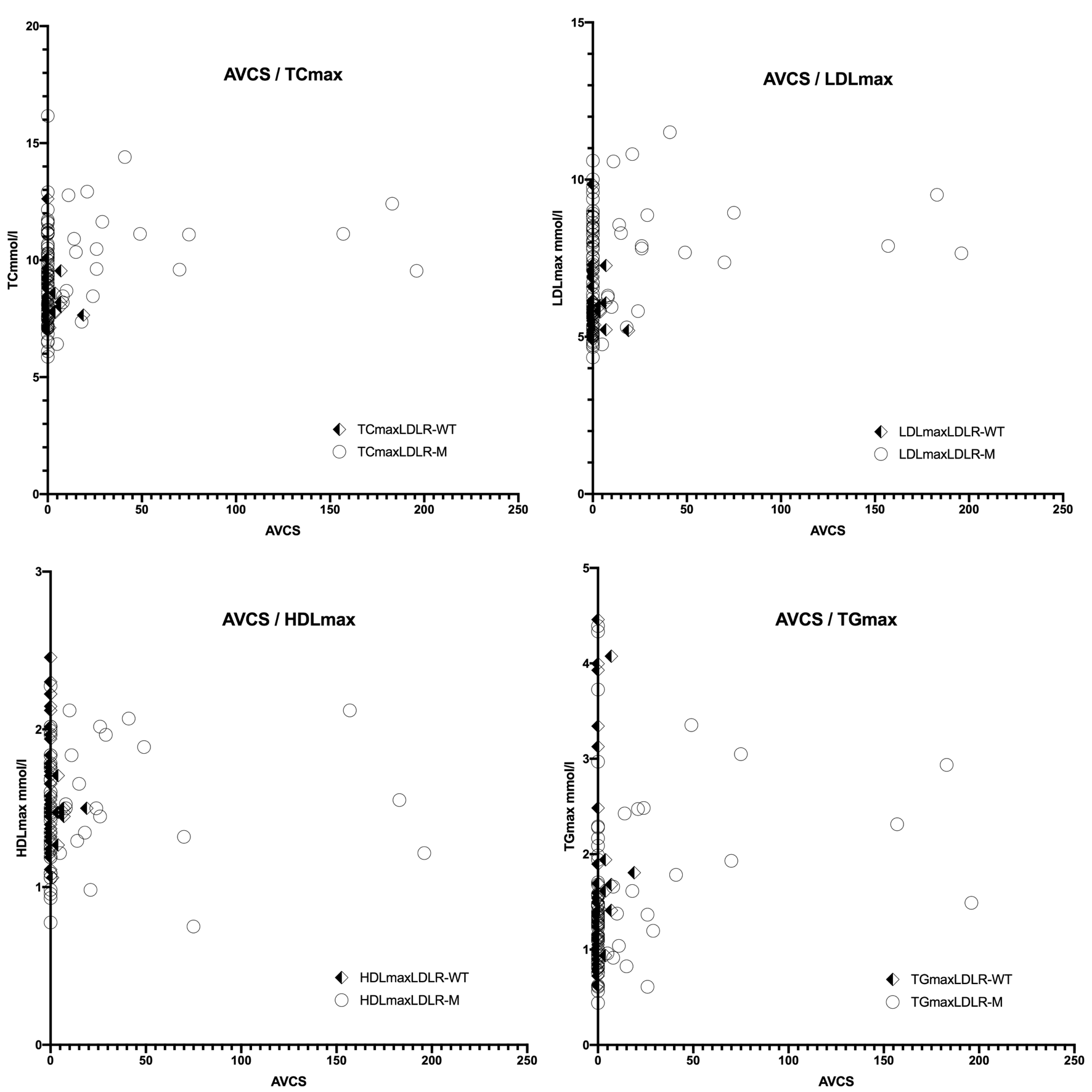

Supplement: S2 Fig — Abbreviations: AVCS—Aortic valve calcium score, LDLR-M–patients with hypercholesterolemia and confirmed LDLR mutation, LDLR-WT (LDLR-wild type)–patients with hypercholesterolemia and nonconfirmed LDLR-mutation, TCmax–total maximum cholesterol level without pharmacotherapy, LDLmax—low‐density lipoprotein, HDLmax—high‐density lipoprotein cholesterol, TGmax–triglycerides. (TIFF) [file pone.0209229.s002.tiff]
