# Supplementary material for: Aortic valve calcium score in hypercholesterolemic patients with and without low-density lipoprotein receptor gene mutation
Source: PLoS One. 2018 Dec 28;13(12):e0209229. doi: 10.1371/journal.pone.0209229 (PMC6310281; doi:10.1371/journal.pone.0209229)
Supplement: S1 Table — Abbreviations: LDLR-M–patients with hypercholesterolemia and confirmed LDLR mutation, NS–not significant, BMI—body mass index, TC and TCmax–total cholesterol level during inclusion to the study and maximum level without pharmacotherapy (same values for patients without prior lipid lowering treatment), LDL and LDLmax—low‐density lipoprotein, HDL and HDLmax—high‐density lipoprotein cholesterol, TG and TGmax–triglycerides, TYCscore–total cholesterol year score, SBP–systolic blood pressure. DBP–diastolic blood pressure. atreatment with insulin or oral anti-diabetic medicine. bsmoking—ever, > 1 pack-year, AVCS—Aortic valve calcium score. (DOCX) [file pone.0209229.s003.docx]

**S3 Table. Clinical characteristics of high and non-high AVSCS subgroups**

|  | High AVCS  (N=24) | Non-high AVCS  (N=98) | | p |
| --- | --- | --- | --- | --- |
| Age (years) | 58.8 ± 9.5 | 48 ± 10.5 | | <0.001 |
| Gender | 12M, 12 F | 44M, 54F | | ns |
| LDLR-M patients | 20 (83%) | 52 (53%) | | <0.05 |
| BMI (kg/m^2^) | 26.7 ± 3.7 | 26.6 ± 4.3 | | ns |
| TC max (mmol/L) | 10 ± 2 | 8.8 ± 1.7 | | <0.01 |
| LDL max (mmol/L) | 7.6 ± 1.9 | 6.5 ± 1.7 | | <0.01 |
| HDL max (mmol/L) | 1.5 ± 0.3 | 1.5 ± 0.3 | | ns |
| TG max (mmol/L) | 1.9 ± 0.9 | 1.5 ± 0.9 | | <0.01 |
| TC (mmol/L) | 7.8 ± 2.4 | 7.3 ± 1.9 | | ns |
| LDL (mmol/L) | 5.7 ± 2.2 | 5.2 ± 1.7 | | ns |
| HDL (mmol/L) | 1.5 ± 0.3 | 1.5 ± 0.3 | | ns |
| TG (mmol/L) | 1.4 ± 0.5 | 1.5 ± 0.8 | | ns |
| TCY score (mmol-year/L) | 538.1±155.9 | 414.8±114.8 | <0.001 | |
| SBP (mmHg) | 140.5± 17,1 | 130.8 ± 13.9 | | <0.05 |
| DBP (mmHg) | 87.3 ± 9.8 | 82 ± 10 | | <0.05 |
| Diabetes^a^ (n) | 1 (1.4%) | 5 (10%) | | ns |
| Smoking^b^ (n) | 2 (8.3%) | 37 (4.1%) | | ns |
| Statin treatment on 1^st^ visit (n) | 14 (58.3%) | 42 (42.8%) | | ns |
